# Anesthesia Type and Outcomes After Transfemoral TAVI: A Time-Sensitive Comparative Analysis

**DOI:** 10.3390/life16040584

**Published:** 2026-04-01

**Authors:** Tuncay Kiris, Fatma Esin, Hakan Bozkurt, Berkay Palac, Bahadır Akar, Aykan Celik, Emre Özdemir, Murat Aksun, Mustafa Karaca

**Affiliations:** 1 Department of Cardiology, Izmir Katip Çelebi University, Atatürk Training and Research Hospital, Izmir 35360, Turkey; 2 Department of Anesthesiology, Izmir Katip Çelebi University, Atatürk Training and Research Hospital, Izmir 35360, Turkey

**Keywords:** TAVI, anesthesia strategy, local anesthesia, conscious sedation, general anesthesia, win-ratio, restricted mean survival time, time-dependent ROC, MACCE

## Abstract

**Background:** The optimal anesthesia strategy for transfemoral transcatheter aortic valve implantation (TAVI) remains uncertain. We evaluated the impact of local anesthesia, conscious sedation, and general anesthesia on early and long-term outcomes after TAVI. Methods: This single-center cohort included 401 patients undergoing transfemoral TAVI with local anesthesia (LA, *n* = 77), conscious sedation (CS, *n* = 147), or general anesthesia (GA, *n* = 177). Outcomes were assessed using hierarchical win-ratio analysis prioritizing mortality over major adverse cardiovascular and cerebrovascular events (MACCE), supported by Kaplan–Meier and restricted mean survival time analyses. Sensitivity analyses using inverse probability of treatment weighting (IPTW) were performed to account for baseline differences between groups. **Results:** Baseline comorbidities were broadly comparable, although GA patients had higher-risk anatomical and procedural features. In unadjusted win-ratio analyses, LA showed a significant advantage over GA at 0–6 months (win ratio [WR] 1.79; 95% CI 1.10–2.93; *p* = 0.020). After multivariable adjustment, LA remained superior to GA at 6–12 and 12–24 months (adjusted WR 1.67 and 1.56, both *p* < 0.05). One-year mortality differed significantly among groups (*p* = 0.012). RMST analysis demonstrated a cumulative survival advantage for LA versus GA, reaching 6.6 months at 60 months. MACCE-free survival was largely comparable across strategies. However, in IPTW-weighted analyses, anesthesia type was not independently associated with mortality or MACCE. **Conclusions:** Minimally invasive anesthesia strategies were associated with more favorable early survival patterns after transfemoral TAVI in primary analyses. However, after adjustment for baseline differences using IPTW, anesthesia type was not independently associated with mortality or MACCE. These findings suggest that apparent outcome differences may partly reflect underlying patient risk profiles rather than a purely causal effect of anesthesia strategy.

## 1. Introduction

Severe aortic stenosis is a progressive, life-threatening condition, and transfemoral TAVI has become an established treatment option across all surgical risk categories [1,2,3]. With the widespread adoption of TAVI, attention has increasingly focused on optimizing peri-procedural management, among which anesthesia strategy is a key modifiable factor [4,5,6].

Historically, TAVI was performed under GA to ensure procedural control and hemodynamic stability [7]. However, emerging evidence suggests that GA may be associated with greater hemodynamic variability, higher vasoactive support, longer procedures, and prolonged intensive care and hospital stay, while minimalist strategies using CS or LA may mitigate physiological stress and streamline care [8,9,10,11,12,13]. Several observational studies and meta-analyses have reported reduced complications, shorter hospitalization, and potentially lower early mortality with less invasive anesthesia compared with GA, and randomized data (e.g., SOLVE-TAVI) have demonstrated non-inferiority of CS relative to GA for early outcomes [14,15,16,17].

Nonetheless, most prior work has focused on short-term outcomes, with limited and inconsistent data regarding the long-term effects of anesthesia type on mortality and MACCE, particularly when comparing LA directly with both CS and GA [18,19]. Moreover, many studies evaluating outcomes after TAVI have relied primarily on Cox regression and conventional Kaplan–Meier analyses, which may be suboptimal in the presence of non-proportional hazards and do not account for the clinical priority of mortality over other events. [20,21].

To address these gaps, the present study examined the association between anesthesia strategy and long-term mortality and MACCE in a large real-world transfemoral TAVI cohort using a comprehensive set of time-to-event techniques, including hierarchical win-ratio analysis prioritizing mortality over MACCE, landmark Kaplan–Meier curves, RMST, and time-dependent ROC modelling.

## 2. Methods

### 2.1. Study Design and Population

**Figure 1:**
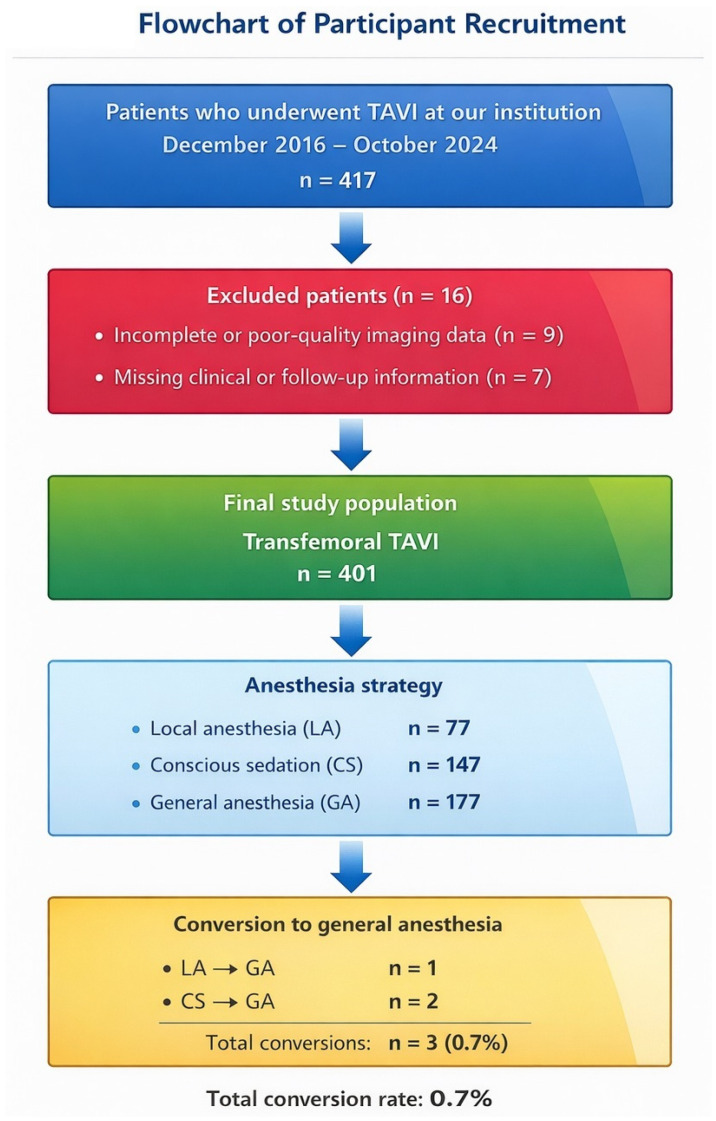
Flowchart of Participint Recruitment.

A total of 417 consecutive patients who underwent transfemoral TAVI between December 2016 and October 2024 at a tertiary care center with a dedicated structural heart team were initially screened. Patients were excluded if they had incomplete or poor-quality imaging data (*n* = 9) or missing clinical or follow-up information (*n* = 7). The final study population consisted of 401 patients (Figure 1). All procedures were performed by experienced operators following contemporary guideline-directed indications and local heart-team decisions regarding device selection and access strategy. A subset of this cohort had been reported previously with shorter follow-up [22]; the present analysis re-evaluated the entire population with extended follow-up and newly defined MACCE components, enabling a broader and methodologically distinct assessment.

### 2.2. Anesthesia Strategies

Patients were categorized into three groups according to the anesthesia technique used during TAVI:

• Local anesthesia (LA): Vascular access obtained under ultrasound guidance; percutaneous femoral local infiltration with 1–2% lidocaine; no systemic sedatives; patients remained fully awake with spontaneous breathing.
• Conscious sedation (CS): Mild-to-moderate sedation using titrated midazolam, fentanyl, or dexmedetomidine at the anesthesiologist’s discretion; patients maintained spontaneous respiration and verbal responsiveness without airway devices.
• General anesthesia (GA): Induction with propofol and/or etomidate followed by neuromuscular blockade, endotracheal intubation, and mechanical ventilation; hemodynamics managed by a dedicated cardiac anesthesia team including vasoactive support as needed.

The choice of anesthesia strategy was not randomized. The decision was made by the multidisciplinary heart team in collaboration with the interventional cardiologist and anesthesiologist according to individual clinical characteristics. Factors influencing the choice of anesthesia included frailty status, comorbidity burden, respiratory reserve, hemodynamic stability, anticipated procedural complexity, patient cooperation, and the potential need for advanced airway management or intraprocedural imaging support. Therefore, anesthesia allocation was individualized based on clinical judgment.

All procedures were performed in the catheterization laboratory by a multidisciplinary heart team. Although a hybrid operating room was not routinely used, cardiac surgical backup and anesthesiology support were available within the institution in case of emergency conversion or procedural complications. Procedures performed under local anesthesia followed a minimalist TAVI approach. An anesthesiologist was not routinely present in the catheterization laboratory; however, patients were continuously monitored for hemodynamic and respiratory parameters by the procedural team. Local anesthesia was achieved with lidocaine infiltration at the vascular access site. An anesthesiology support team and equipment for rapid conversion to general anesthesia were available if required. In the conscious sedation group, sedation depth was assessed clinically rather than by processed EEG monitoring. Sedative agents were administered in a titrated manner to maintain patient comfort while avoiding deep sedation, and oxygen saturation, respiratory status, and hemodynamic parameters were continuously monitored during the procedure. In the general anesthesia group, transesophageal echocardiography was used at the operator’s discretion for intraprocedural imaging guidance. All procedures were performed with continuous invasive arterial pressure monitoring and echocardiographic guidance, and standardized postoperative care included telemetry for at least 48 h and pre-discharge transthoracic echocardiography.

### 2.3. Study Endpoints

The primary endpoint was a hierarchical composite outcome evaluated using the win-ratio method, with all-cause mortality designated as the highest-priority event and MACCE as the second level. For each patient pair, time to death was compared first; if both patients were alive or died at the same time, the comparison advanced to the occurrence and timing of MACCE. Clinical follow-up was performed through outpatient visits and review of hospital records. Overall, all-cause mortality and MACCE were assessed during follow-up of up to 103 months after the index TAVI procedure.

MACCE was defined to align with contemporary Valve Academic Research Consortium-3 (VARC-3) and related criteria and included [23]:

• Stroke (ischemic or hemorrhagic) with clinical symptoms and imaging confirmation
• Myocardial infarction per the Fourth Universal Definition
• Coronary revascularization (PCI or CABG)
• Hospitalization for heart failure, based on clinical, biomarker, and/or echocardiographic evidence
• Clinically significant arrhythmia (new-onset atrial fibrillation, sustained ventricular tachycardia/fibrillation, or high-grade atrioventricular block requiring permanent pacemaker implantation)
• Bioprosthetic valve dysfunction (structural or non-structural deterioration, progression of paravalvular leak, or valve thrombosis with hemodynamic or embolic impact)

Secondary endpoints included the individual MACCE components, overall mortality at different time points, and RMST-based comparisons of survival and MACCE-free survival.

### 2.4. Statistical Analysis

Continuous variables were summarized as mean ± standard deviation or median (interquartile range) according to the Shapiro–Wilk test; group differences were assessed using one-way ANOVA or Kruskal–Wallis tests, and categorical variables with chi-square or Fisher’s exact tests. Long-term all-cause mortality and MACCE were evaluated using Kaplan–Meier curves with log-rank tests across the three anesthesia groups. Landmark analyses at 30 days, 12 months, and 24 months were performed to explore temporal variation in relative risks and to distinguish early from late divergences. Because proportional hazards assumptions may be violated in TAVI populations and effects can accrue gradually over time, RMST was calculated at 3- and 5-year horizons as a robust, model-independent estimate of average event-free time. Differences in RMST (ΔRMST) between anesthesia groups were reported as clinically interpretable measures of long-term benefit or disadvantage. A hierarchical win-ratio framework was applied to incorporate the clinical priority of mortality over MACCE and provide a more nuanced assessment beyond conventional time-to-first-event approaches. Pairwise comparisons among the three anesthesia strategies (LA vs. CS, LA vs. GA, CS vs. GA) were performed by counting wins and losses based on the ordered time-to-event hierarchy. Both unadjusted and adjusted win-ratio analyses were conducted. Adjusted models included age, left ventricular ejection fraction, estimated glomerular filtration rate, and valve type, using a Cox-based stratified win-ratio approach. Because multiple time windows and pairwise comparisons were examined, these analyses should be considered exploratory and hypothesis-generating, and the results interpreted with appropriate caution.

Two complementary win-ratio strategies were implemented:

1. **Time-varying win ratio**: Estimated via sliding time windows across follow-up, generating dynamic curves illustrating how relative advantages between groups evolved over time.
2. **Time-stratified win ratio**: Calculated within prespecified intervals (0–6, 6–12, 12–24, 24–36, 36–60, and >60 months), with 95% CIs obtained by non-parametric bootstrap resampling (1000 iterations). Win-ratio analyses were performed using a hierarchical comparison framework prioritizing all-cause mortality over MACCE. Patients in the comparison groups were evaluated using pairwise comparisons across all possible patient pairs. For each pair, outcomes were assessed according to the predefined hierarchy, with a “win” assigned to the patient experiencing the more favorable outcome. If both patients experienced the same event type at the same time or if neither experienced the event during the comparison interval, the pair was classified as a tie. Censoring was handled according to standard win-ratio methodology, with comparisons limited to available follow-up time for each patient pair.

Because anesthesia allocation was not randomized, a propensity score–based analysis using inverse probability of treatment weighting (IPTW) was performed to mitigate potential confounding. The propensity score model incorporated clinically relevant baseline variables potentially influencing anesthesia selection and outcomes, including age, sex, smoking status, peripheral arterial disease, chronic obstructive pulmonary disease, previous PCI, left ventricular ejection fraction, systolic pulmonary artery pressure, estimated glomerular filtration rate, valve type, moderate-to-severe mitral regurgitation, pleural effusion, EuroSCORE II, and procedural era. Covariate balance before and after weighting was assessed using standardized mean differences (SMD), with values < 0.1 indicating good balance. IPTW-weighted Cox proportional hazards models were then applied to evaluate the association between anesthesia strategy and mortality and MACCE.

Conventional and time-dependent ROC analyses were used to evaluate whether inclusion of anesthesia type improved discrimination for mortality and MACCE beyond a clinical baseline model. All statistical analyses were performed using SPSS software version 30.0 (IBM Corp., Armonk, NY, USA) and R software version 4.5.1 (R Foundation for Statistical Computing, Vienna, Austria), with two-sided *p* < 0.05 considered statistically significant.

## 3. Results

### 3.1. Baseline Characteristics

A total of 401 patients were included: 77 in the LA group, 147 in the CS group, and 177 in the GA group, with a median follow-up of 26.6 months (IQR 14.88–41.41). Age (median 75–77 years) and sex distribution were similar between groups, as were most cardiovascular risk factors, although dyslipidemia was more frequent in LA and smoking was less prevalent in GA (Table 1). Peripheral vascular disease increased stepwise from LA to GA (7% vs. 21%; *p* = 0.001, Table 1), and GA patients more often had moderate-to-severe mitral regurgitation and other high-risk valvular features.

Baseline surgical risk assessed by the EuroSCORE II differed significantly among the anesthesia groups. Median EuroSCORE II values were 2.20 (IQR 1.69–3.28) in the LA group, 2.83 (2.12–4.01) in the CS group, and 4.48 (2.63–7.40) in the GA group (*p* < 0.001). These findings indicate that patients managed under general anesthesia had a substantially higher preprocedural operative risk (Table 1).

Procedurally, self-expanding valves predominated in LA and GA, whereas balloon-expandable valves were more common in CS (Table 1). Temporary pacing requirements were significantly higher in CS and GA than in LA, while permanent pacemaker implantation did not differ. Preoperative pleural effusion and early MACCE were more frequent in GA, and overall all-cause mortality increased progressively from LA (27%) to CS (35%) and GA (49%; *p* = 0.003, Table 1).

Baseline laboratory parameters and echocardiographic valve gradients were comparable among the three anesthesia groups; however, systolic pulmonary artery pressure was significantly higher in the intubation group compared with local anesthesia and conscious sedation groups (*p* = 0.013, Table 2), while all other variables showed no statistically significant differences (Table 2). During the procedure, conversion to general anesthesia occurred in 3 patients (0.75%), including two patients initially treated under conscious sedation and one patient under local anesthesia.

### 3.2. Hierarchical Win-Ratio Analysis

**Figure 2:**
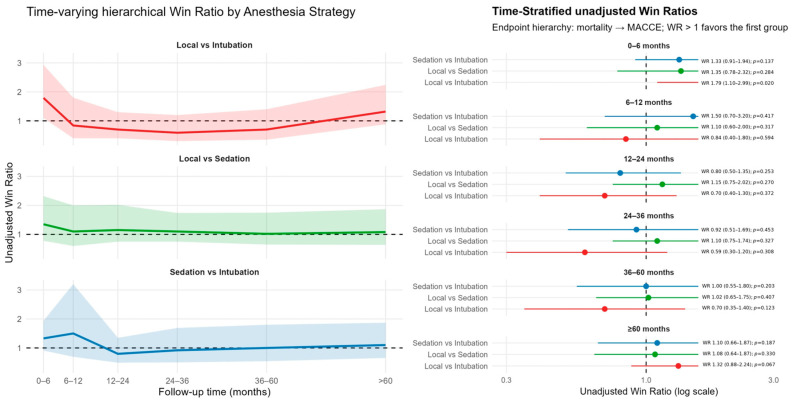
Time-varying hierarchical unadjusted win ratio by anesthesia type.

In unadjusted, time-stratified hierarchical win-ratio analyses, anesthesia technique significantly influenced early outcomes. During the first 0–6 months, LA demonstrated clear superiority over GA (WR 1.79; 95% CI 1.10–2.93; *p* = 0.020, Figure 2), indicating more favorable results with respect to the mortality-first hierarchy. LA versus CS and CS versus GA yielded WRs > 1 (1.35 and 1.33, respectively), but these did not reach statistical significance.

Between 6–12 and 12–24 months, unadjusted WRs across all pairwise comparisons approximated unity, suggesting no strong mid-term differential effect of anesthesia on the hierarchical composite. Beyond 24 months, WRs remained largely neutral with wide CIs overlapping 1, consistent with an absence of sustained long-term differences between strategies.

**Figure 3:**
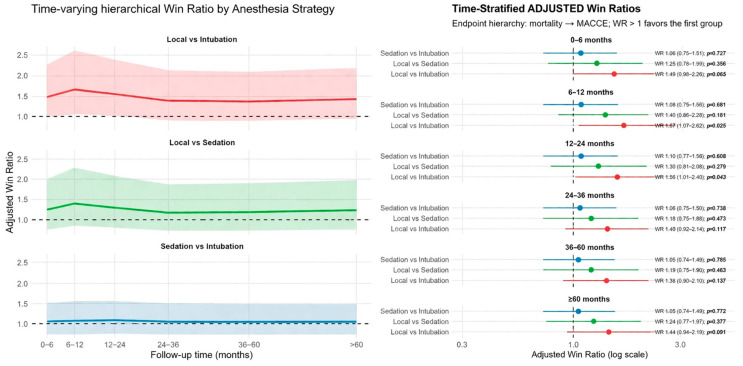
Time-varying hierarchical adjusted win ratio by anesthesia type.

After multivariable adjustment, during the 0–6 month period, the adjusted LA vs. GA win ratio was higher in the LA group, although the difference did not reach statistical significance (WR 1.49; 95% CI 0.98–2.26; *p* = 0.065; Figure 3). However, LA showed statistically significant superiority over GA at 6–12 months (WR 1.67; 95% CI 1.07–2.62; *p* = 0.025, Figure 3) and again at 12–24 months (WR 1.56; 95% CI 1.01–2.40; *p* = 0.043, Figure 3), while comparisons involving CS remained neutral. After 24 months, adjusted WRs for all anesthesia comparisons converged around 1, with no late differences detected (Figure 3).

These findings indicate that LA may confer a transient, mainly early- to mid-term therapeutic advantage over GA for the prioritized composite of mortality and MACCE, whereas no anesthesia technique maintains a durable hierarchical benefit beyond approximately two years.

### 3.3. Kaplan–Meier Survival Analyses

**Figure 4:**
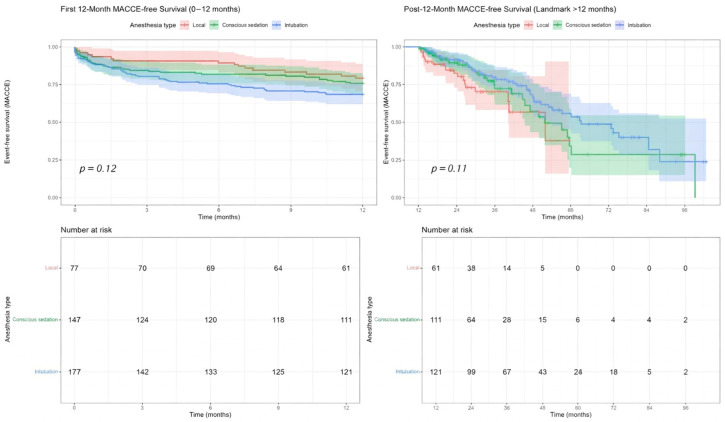
Landmark analysis in the periods of 0–12 months and after the 12th month for major adverse cardiovascular and cerebrovascular events (MACCE).

For MACCE-free survival, no statistically significant variation was observed among groups at 0–12 months (*p* = 0.12, Figure 4), despite numerically more favorable early profiles for LA and CS versus GA. Post-12-month landmark curves remained broadly similar (*p* = 0.11, Figure 4), and apparent late divergences were limited by smaller numbers at risk and wide CIs, supporting an overall neutral long-term effect of anesthesia type on MACCE-free survival.

**Figure 5:**
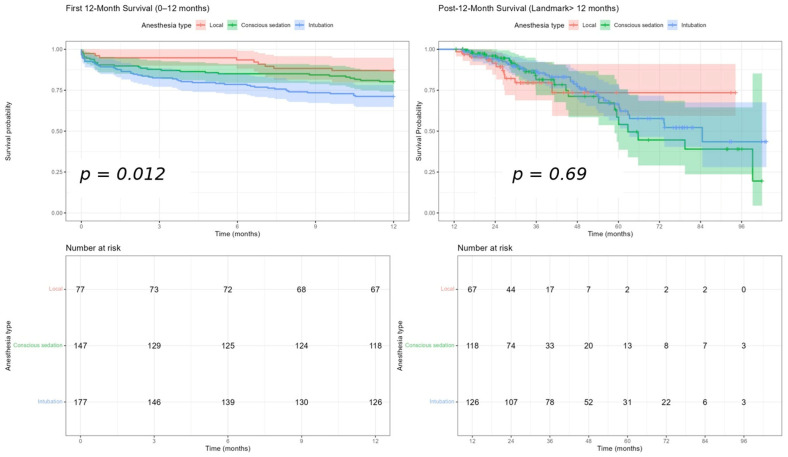
Landmark analysis in the periods of 0–1 month, and after the 12th month for mortality.

In the first 12 months after TAVI, overall survival differed significantly among anesthesia groups (log-rank *p* = 0.012, Figure 5), with LA exhibiting the most favorable early survival, followed by CS and then GA. Landmark analysis beyond 12 months showed no significant survival differences (*p* = 0.69, Figure 5), as curves converged and overlapped, consistent with the win-ratio results indicating diminishing anesthesia-related effects over time.

### 3.4. RMST Analysis

**Figure 6:**
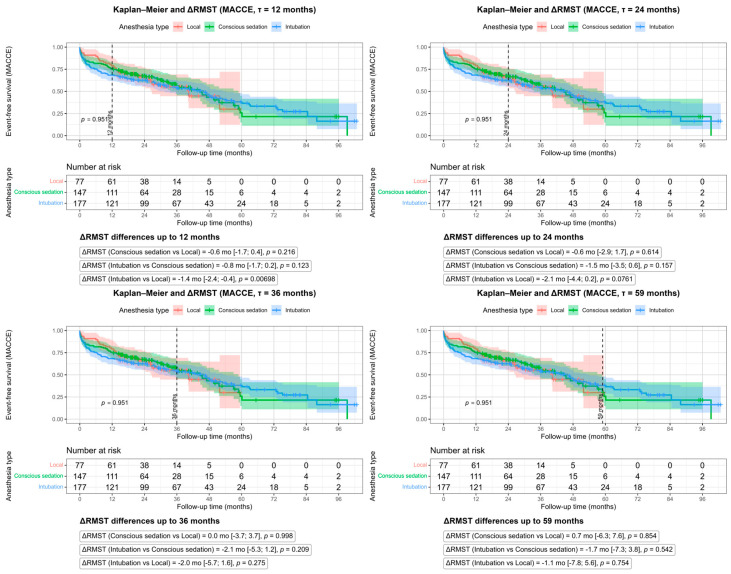
Restricted Mean Survival Time (RMST) analysis for MACCE in 12–24–36–59–60 months.

MACCE-free RMST did not differ meaningfully between groups over long-term follow-up, although GA was associated with a short-term disadvantage compared with LA at 12 months (ΔRMST −1.4 months; 95% CI −2.4 to −0.4; *p* = 0.007, Figure 6).

**Figure 7:**
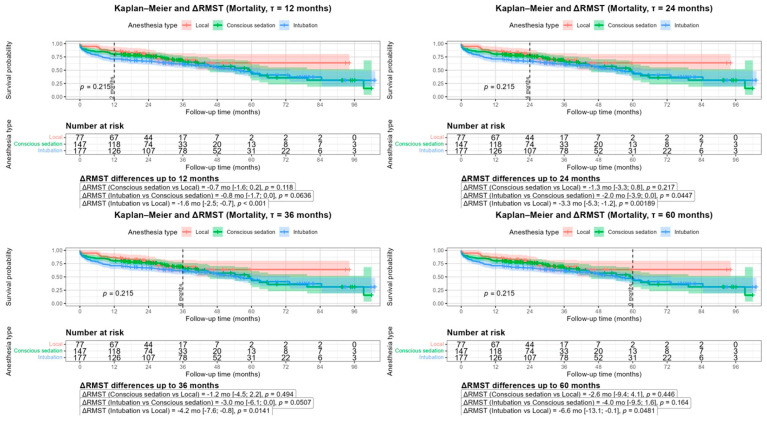
Restricted Mean Survival Time (RMST) analysis for mortality in 12, 24–36–60 months.

RMST analyses at 12, 24, 36, and 60 months showed that GA was consistently associated with poorer survival compared with LA, with ΔRMST ranging from −1.6 months at 12 months to −6.6 months at 60 months (all *p* < 0.05, Figure 7). Survival differences between CS and LA were small and non-significant, whereas GA showed a tendency toward reduced survival vs. CS, reaching significance at selected time points (e.g., 12 and 24 months, Figure 7).

#### Propensity Score-Based Analysis

**Figure 8:**
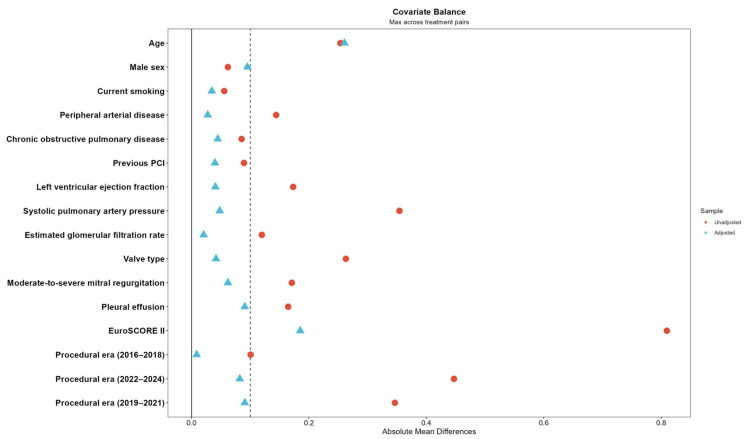
Covariate balance before and after inverse probability of treatment weighting (IPTW). Standardized mean differences (SMDs) for baseline covariates are shown before (red circles) and after (blue triangles) IPTW weighting across anesthesia groups. The vertical dashed line indicates the conventional threshold for acceptable balance (SMD = 0.1). After weighting, covariate balance improved substantially, with most standardized mean differences approaching or below the conventional threshold of 0.1.

To further evaluate the potential impact of baseline risk imbalance, IPTW-weighted Cox proportional hazards models were performed. After weighting, covariate balance improved substantially, with all standardized mean differences below 0.2, with the majority below the conventional threshold of 0.1. (Table 3, Figure 8). In the IPTW-weighted analysis, anesthesia type was not independently associated with all-cause mortality (global Wald *p* = 0.50) or MACCE (global Wald *p* = 0.60). Compared with local anesthesia, the adjusted hazard ratios for mortality were 1.42 (95% CI 0.74–2.73) for conscious sedation and 1.39 (95% CI 0.74–2.61) for general anesthesia. For MACCE, the corresponding hazard ratios were 1.03 (95% CI 0.64–1.67) and 0.87 (95% CI 0.55–1.40), respectively.

### 3.5. ROC Analyses

**Figure 9:**
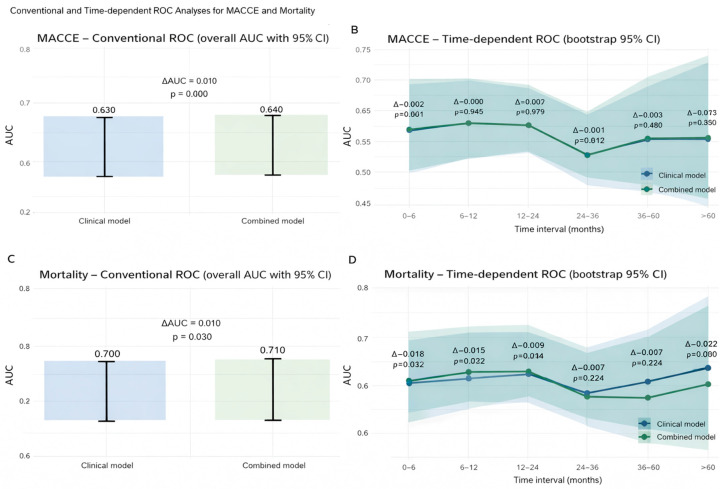
Conventional versus time-dependent ROC analyses of the multivariable model and multivariable model plus anesthesia type. (**A**) Conventional ROC analysis for MACCE. (**B**) Time-dependent ROC analysis for MACCE. (**C**) Conventional ROC analysis for mortality. (**D**) Time-dependent ROC analysis for mortality.

Standard ROC analysis demonstrated that adding anesthesia type to a baseline clinical model did not significantly improve discrimination for MACCE (ΔAUC 0.010; *p* = 0.400, Figure 9A), and time-dependent ROC curves similarly showed negligible incremental value for MACCE prediction across follow-up (Figure 9B).

In contrast, mortality discrimination improved modestly but significantly with the inclusion of anesthesia type (AUC 0.700 vs. 0.710; ΔAUC 0.010; *p* = 0.030, Figure 9C). Time-dependent ROC confirmed this pattern, with higher AUCs for the combined model at 0–6 months (ΔAUC 0.018; *p* = 0.016, Figure 9D) and 6–12 months (ΔAUC 0.015; *p* = 0.032, Figure 9D), but not at 12–24 or 24–36 months; a small late divergence re-emerged beyond 60 months (Figure 9D).

## 4. Discussion

In this study, local anesthesia appeared to be associated with more favorable early survival patterns compared with general anesthesia in primary hierarchical win-ratio analyses. However, when baseline differences between groups were addressed using propensity score–based inverse probability of treatment weighting (IPTW), anesthesia type was not independently associated with mortality or MACCE. These findings suggest that part of the survival differences observed in the primary analyses may have been influenced by baseline risk imbalance between anesthesia groups rather than representing a purely causal effect of anesthesia strategy.

These findings augment and partially extend the existing corpus of information. Prior observational studies and comprehensive national registries have consistently shown that less invasive anesthesia, particularly conscious sedation, provides advantages in terms of peri-procedural stability, reduced hospital stays, and lower early mortality rates compared to general anesthesia [14,16,17,18]. Nonetheless, research findings on mid- to long-term outcomes have been inconsistent, and there is a scarcity of data comparing local anesthetics specifically with both sedation and intubation [20,21]. Many previous studies in the TAVI literature have relied primarily on Cox regression and conventional Kaplan–Meier analysis, which may be suboptimal in the presence of non-proportional hazards and do not account for the clinical hierarchy between mortality and other outcomes [20,21,22,23].

Our analysis enriches this literature by utilizing diverse complementary and methodologically robust statistical techniques, including hierarchical win-ratio analysis, landmark and time-varying Kaplan–Meier curves, restricted mean survival time (RMST), and both conventional and time-dependent ROC modeling [24,25,26]. These techniques allowed us to distinguish between short-term and long-term effects, quantify initial survival advantages, assess cumulative survival discrepancies despite non-proportional hazards, and comprehensively evaluate the supplementary prognostic relevance of anesthetic type. This thorough analytical method offers a more nuanced and timely understanding of the influence of anesthetic type on TAVI outcomes than prior studies have accomplished. An apparent difference between the RMST and hierarchical win-ratio findings should also be considered. While RMST analyses suggested a persistent survival disadvantage associated with general anesthesia up to 60 months, the win-ratio analysis indicated attenuation of the differences beyond approximately two years. This discrepancy likely reflects methodological differences between the two approaches. The hierarchical win-ratio prioritizes early clinical events within a composite hierarchy, whereas RMST summarizes cumulative survival time across the entire follow-up period. As a result, RMST may capture longer-term survival differences that are less apparent in hierarchical composite analyses. As illustrated by the numbers at risk in the landmark survival analyses, the number of patients at risk decreases substantially at later follow-up time points. Therefore, the RMST difference observed at 60 months likely reflects early divergence in survival rather than a sustained separation of hazards over long-term follow-up.

Baseline characteristics also suggested that patients treated under general anesthesia had a higher underlying risk profile, as reflected by the greater prevalence of peripheral vascular disease and moderate-to-severe mitral regurgitation. In addition, baseline EuroSCORE II values were significantly higher in the GA group compared with the LA and CS groups. This suggests that general anesthesia was more frequently selected in patients with higher clinical risk or anticipated procedural complexity. Consequently, part of the observed differences in early outcomes may reflect underlying risk profiles rather than a purely independent effect of the anesthetic strategy. To address this potential imbalance, a propensity score-based IPTW analysis was performed. After weighting, baseline characteristics between the anesthesia groups were well balanced, and anesthesia type was not independently associated with mortality or MACCE in IPTW-weighted Cox models. These findings suggest that the differences observed in unadjusted analyses were likely influenced, at least in part, by baseline risk imbalance between the anesthesia groups. The observed superiority of local anesthesia and conscious sedation may be explained by a combination of physiologic and procedural mechanisms, including greater hemodynamic stability through avoidance of general anesthesia–induced vasoplegia, hypotension, catecholamine surges, and myocardial oxygen imbalance—particularly detrimental in patients with severe aortic stenosis [27,28,29,30,31]; reduced respiratory complications due to elimination of mechanical ventilation–associated risks such as postoperative pneumonia, atelectasis, and hypoxemia, all of which have been linked to increased mortality after TAVI [32,33]; attenuation of the systemic inflammatory and neurohumoral stress response typically triggered by general anesthesia and known to exacerbate myocardial dysfunction and renal injury [34]; and more favorable procedural conditions that facilitate earlier mobilization and are associated with lower rates of conduction disturbances, as reflected by the markedly higher temporary pacing requirements observed in patients undergoing sedation or intubation [35,36].

Our findings support a procedural approach that prioritizes minimally invasive anesthetic methods whenever feasible. Local anesthetic is particularly advantageous for certain patients, while conscious sedation provides a safe intermediate alternative. Intubation should be restricted to circumstances requiring airway protection, hemodynamic stabilization, complex anatomical factors, or anticipated procedural difficulties; however, physicians must recognize the potential survival benefits and detriments associated with its use. These findings highlight the importance of accounting for baseline risk differences when interpreting observational comparisons of anesthesia strategies in TAVI.

This study has several important limitations that should be considered when interpreting the findings. The study’s retrospective, non-randomized design introduces the possibility of selection bias, as anesthesia was chosen based on operator preference, patient characteristics, and procedural complexity, despite multivariable adjustment. Although a propensity score–based IPTW analysis was performed to reduce baseline imbalances between anesthesia groups, residual confounding due to unmeasured variables cannot be completely excluded. Also, baseline operative risk differed between groups, with higher EuroSCORE II values observed in the GA group, suggesting that anesthesia selection may have been influenced by underlying patient risk and anticipated procedural complexity. In addition, confounding by indication and temporal practice changes may have influenced the observed associations. In the early phases of TAVI adoption, general anesthesia was more commonly used, whereas less invasive strategies such as conscious sedation and local anesthesia became increasingly adopted as operator experience and procedural confidence increased. Consequently, patients treated with local anesthesia may have represented a lower-risk population, and improvements in operator experience, device technology, and procedural techniques over time may also have contributed to the observed outcome differences. Residual confounding—such as frailty, pulmonary reserve, anesthesiologist experience, and detailed hemodynamics—cannot be fully excluded. The absence of routine processed EEG monitoring in the conscious sedation group may have limited objective assessment of sedation depth. Although sedation was clinically titrated under continuous respiratory and hemodynamic monitoring, transient oversedation cannot be completely excluded. In addition, potential differences in the prevalence of preoperative pleural effusion between the anesthesia groups should be considered. As previously reported by our group [22], preoperative pleural effusion may be associated with adverse outcomes following TAVI. Therefore, residual confounding related to this variable cannot be completely excluded. The LA cohort was smaller than the CS and GA cohorts, potentially limiting power for some comparisons, and temporal changes in devices, techniques, and patient selection may also have influenced outcomes. Late survival estimates were constrained by diminishing numbers at risk. Finally, as an observational analysis, the results should be regarded as hypothesis-generating rather than definitive confirmation of causality. Because multiple time intervals and pairwise comparisons were evaluated, the findings should be interpreted as exploratory and hypothesis-generating.

## 5. Conclusions

In this real-world cohort of patients undergoing transfemoral TAVI, minimally invasive anesthesia strategies were associated with more favorable early survival patterns compared with general anesthesia in primary analyses. However, after adjustment for baseline differences using propensity score–based IPTW, anesthesia type was not independently associated with mortality or MACCE. These findings suggest that the observed outcome differences may partly reflect underlying patient risk profiles rather than a purely causal effect of anesthesia strategy. Further prospective studies are needed to clarify the optimal anesthetic approach in TAVI.

## Figures and Tables

**Table 1:** Clinical and Demographic Findings by Anesthesia Type.

Variables	Local (*n* = 77)	Conscious Sedation (*n* = 147)	Intubation (*n* = 177)	*p*
Age	75.0 ± 7.9	76.9 ± 6.6	77.0 ± 9.0	0.149
Male gender *n* (%)	40 (52)	76 (52)	81 (46)	0.488
Diabetes Mellitus *n* (%)	30 (39)	42 (29)	63 (36)	0.227
Hypertension *n* (%)	57 (74)	98 (67)	113 (64)	0.285
Dyslipidemia *n* (%)	32 (42)	52 (35)	48 (27)	0.058
Carotid Artery Disease *n* (%)	3 (4)	2 (1)	2 (1)	0.273
Smoking *n* (%)	3 (4)	9 (6)	1 (1)	0.018
Coronary Artery Disease *n* (%)	60 (78)	125 (85)	148 (84)	0.389
History of Valve Surgery *n* (%)	2 (3)	3 (2)	4 (2)	0.965
Peripheral Arterial Disease *n* (%)	5 (7%)	14 (10)	37 (21)	0.001
Heart Failure *n* (%)	19 (25)	32 (22)	57 (32)	0.096
COPD *n* (%)	6 (8)	20 (14)	9 (5)	0.024
Previous Stroke *n* (%)	8 (10)	9 (6)	12 (7%)	0.480
Chronic Kidney Disease *n* (%)	20 (26)	40 (27)	48 (27)	0.978
Atrial Fibrillation *n* (%)	18 (23)	33 (22)	51 (29)	0.381
Previous PCI *n* (%)	4 (5)	11 (8)	25 (14)	0.041
LVEF (%)	54.0 ± 10.3	54.8 ± 10.3	53.0 ± 11.4	0.306
EuroSCORE II (median (IQR)	2.20 (1.69–3.28)	2.83 (2.12–4.01)	4.48 (2.63–7.40)	<0.001
Moderate/Severe MR *n* (%)	26 (34)	54 (37)	90 (51)	0.009
Moderate/Severe TR *n* (%)	23 (30)	40 (27)	60 (34)	0.424
Valve Type *n* (%)	1			<0.001
Self-expanding *n* (%)	59 (77)	74 (50)	133 (75)	
Balloon-expandable *n* (%)	18 (23)	73 (50)	44 (25)	
Temporary Pacemaker *n* (%)	13 (17)	71 (48)	82 (46)	<0.001
Permanent Pacemaker *n* (%)	9 (12)	18 (12)	27 (15)	0.643
Pleural Effusion *n* (%)	23 (30)	53 (36)	82 (46)	0.028
MACCE *n* (%)	34 (44)	66 (45)	101 (57)	0.047
Stroke *n* (%)	1(1)	2 (1)	2 (1)	0.982
Clinically significant arrhythmia *n* (%)	0 (0)	7 (5)	5 (3)	0.137
Myocardial infarction *n* (%)	4 (5)	1 (1)	2 (1)	0.035
Coronary revascularization *n* (%)	1 (1)	0 (0)	0 (0)	0.121
Hospitalization for heart failure *n* (%)	4 (5)	13 (9)	14 (8)	0.620
Bioprosthetic valve dysfunction *n* (%)	3 (1)	4 (1)	4 (2)	0.648
Mortality *n* (%)	21 (27)	52 (35)	86 (49)	0.003

Abbreviations: COPD: Chronic Obstructive Pulmonary Disease, PCI: percutaneous coronary intervention, LVEF: left ventricular ejection fraction, MR: Mitral Regurgitation (Moderate/Severe), TR: Tricuspid Regurgitation (Moderate/Severe), MACCE: major adverse cardiovascular and cerebrovascular events. IQR: interquartile range.

**Table 2:** Laboratory Findings by Anesthesia Type.

Variables	Local (*n* = 77)	Conscious Sedation (*n* = 147)	Intubation (*n* = 177)	*p*
HGB (g/dL)	11.1 (1.9)	11.2 (1.6)	11.0 (1.9)	0.468
PLT (×10^3^/μL)	220.8 (70.3)	217.0 (85.9)	217.3 (87.2)	0.940
GFR (mL/min)	73.4 (26.8)	76.9 (29.6)	76.4 (30.4)	0.688
Albumin (g/dL)	37.5 (4.4)	35.2 (7.2)	35.2 (5.3)	0.295
WBC (×10^3^/μL)	7.9 (3.0)	8.1 (3.7)	8.1 (3.3)	0.934
BMI (kg/m^2^)	25.8 (2.1)	28.5 (6.1)	30.9 (5.8)	0.289
Aortic Valve Peak Gradient (mmHg)	74.3 (20.5)	75.8 (22.6)	77.2 (24.0)	0.654
Aortic Valve Mean Gradient (mmHg)	46.3 (14.6)	45.8 (14.8)	46.6 (15.5)	0.898
SPAP (mmHg)	35.8 (13.3)	38.6 (13.9)	40.7 (14.4)	0.013

Abbreviations: HGB: Hemoglobin; PLT: Platelet Count; GFR: Glomerular Filtration Rate; WBC: White Blood Cell Count; BMI: Body Mass Index; SPAP: Systolic Pulmonary Artery Pressure.

**Table 3:** Baseline characteristics before and after inverse probability of treatment weighting (IPTW) by anesthesia group.

Variables	Local (*n* = 77)	Before Conscious Sedation (*n* = 147)	Intubation (*n* = 177)	Local (*n* = 77)	After Conscious Sedation (*n* = 147)	Intubation (*n* = 177)	SMD Before	SMD After
Age (years)	74.96	76.91	76.97	74.42	76.48	76.31	0.254	0.260
Male gender %	51.9	51.7	45.8	45.7	55.3	49.3	0.062	0.095
Smoking %	3.9	6.1	0.6	4.5	3.1	1	0.056	0.034
PAD %	6.5	9.5	20.9	10.5	11.8	13.2	0.144	0.028
COPD %	7.8	13.6	5.1	12.3	8.2	7.9	0.085	0.045
Previous PCI %	5.2	7.5	14.1	4.2	6.3	8.2	0.089	0.040
LVEF(%)	54.03	54.82	52.97	53.02	52.75	52.59	0.173	0.041
SPAP (mmHg)	35.79	38.55	40.7	39.02	39.38	39.68	0.354	0.048
GFR (mL/min)	73.44	76.91	76.36	75.46	75.86	76.05	0.12	0.020
Valve Type %	23.4	49.7	24.9	37.3	33.3	33.1	0.263	0.042
Moderate/Severe MR %	33.8	36.7	50.8	39.8	45.1	46	0.171	0.062
Pleural Effusion %	29.9	36.1	46.3	32.6	41.6	39.9	0.165	0.091
EuroSCORE2	2.93	3.65	6.81	4.24	4.38	5.13	0.81	0.185
Procedural era %								
2016–2018	5.2	15	15.3	13.7	12.9	12.8	0.101	0.009
2019–2021	11.7	12.2	46.3	19.4	27.4	28.5	0.346	0.091
2022–2024	83.1	46.3	38.4	66.9	28.5	58.7	0.447	0.082

Abbreviations: SMD: standardized mean difference, PAD: Peripheral Arterial Disease, GFR: Glomerular Filtration Rate, COPD: Chronic Obstructive Pulmonary Disease, PCI: percutaneous coronary intervention, LVEF: left ventricular ejection fraction, MR: Mitral Regurgitation (Moderate/Severe), SPAP: Systolic Pulmonary Artery Pressure.

## Data Availability

The dataset has been previously partially reported (ref); however, the current analysis includes extended follow-up and newly defined MACCE endpoints that have not been published before. The data presented in this study are available on request from the corresponding author due to privacy concerns.
